# Improving prediction of tacrolimus concentration using a combination of population pharmacokinetic modeling and machine learning in chinese renal transplant recipients

**DOI:** 10.3389/fphar.2024.1389271

**Published:** 2024-05-09

**Authors:** Yu-Ping Wang, Xiao-Ling Lu, Kun Shao, Hao-Qiang Shi, Pei-Jun Zhou, Bing Chen

**Affiliations:** ^1^ Department of Pharmacy, Ruijin Hospital, Shanghai Jiaotong University, School of Medicine, Shanghai, China; ^2^ Center for Organ Transplantation, Ruijin Hospital, Shanghai Jiaotong University, School of Medicine, Shanghai, China

**Keywords:** renal transplant recipients, population pharmacokinetic, machine learning, XGBboost, tacrolimus

## Abstract

**Aims:**

The population pharmacokinetic (PPK) model-based machine learning (ML) approach offers a novel perspective on individual concentration prediction. This study aimed to establish a PPK-based ML model for predicting tacrolimus (TAC) concentrations in Chinese renal transplant recipients.

**Methods:**

Conventional TAC monitoring data from 127 Chinese renal transplant patients were divided into training (80%) and testing (20%) datasets. A PPK model was developed using the training group data. ML models were then established based on individual pharmacokinetic data derived from the PPK basic model. The prediction performances of the PPK-based ML model and Bayesian forecasting approach were compared using data from the test group.

**Results:**

The final PPK model, incorporating hematocrit and *CYP3A5* genotypes as covariates, was successfully established. Individual predictions of TAC using the PPK basic model, postoperative date, *CYP3A5* genotype, and hematocrit showed improved rankings in ML model construction. XGBoost, based on the TAC PPK, exhibited the best prediction performance.

**Conclusion:**

The PPK-based machine learning approach emerges as a superior option for predicting TAC concentrations in Chinese renal transplant recipients.

## 1 Introduction

Tacrolimus (TAC), a calcineurin inhibitor, is widely employed to prevent allograft rejection in transplant recipients following solid organ transplantation (SOT) (Zhang et al., 2017; Yu et al., 2018). TAC has a narrow therapeutic window (Mac Guad et al., 2016). Patients with elevated TAC exposure may experience toxicity, such as neurotoxicity, nephrotoxicity, and post-transplant diabetes mellitus. Conversely, insufficient TAC exposure may be associated with allograft rejection or even graft loss (Yaowakulpatana et al., 2016; Thongprayoon et al., 2020). TAC is rapidly absorbed, with peak blood concentration occurring 0.5–1 h post-administration. Its bioavailability in patients in a steady state is approximately 25% (5%–93%). Absorbed TAC undergoes extensive metabolism by CYP3A4 and CYP3A5 in the gut mucosa and liver, resulting in over ten different metabolites (Zuo et al., 2013b). More than 95% of the metabolites are eliminated through bile (Chitnis et al., 2013; Taber et al., 2021). TAC exhibits variable pharmacokinetic (PK) properties, and even slight dose variations can significantly impact individuals (Teng et al., 2022). Various factors, including genetic polymorphisms, pathophysiological indices, and concomitant drugs, may influence TAC PKs. *CYP3A5* genotype is the most frequently studied factor (Ghafari et al., 2019), accounting for 40%–50% of the variability in TAC clearance (Benkali et al., 2010; Vannaprasaht et al., 2013; Zhang et al., 2013; Cheng et al., 2015; Mac Guad et al., 2016; Tang et al., 2016; Yaowakulpatana et al., 2016; Htun et al., 2018; Uno et al., 2019; Bezerra et al., 2020; Cheung et al., 2020). Additionally, factors such as postoperative date (POD), hematocrit (HCT), body weight, liver function, and the concurrent use of voriconazole or Wuzhi capsules may impact the exposure and dosing regimen of TAC (Degraeve et al., 2020).

Therapeutic drug monitoring (TDM) is commonly employed to ensure optimal exposure to TAC (Vannaprasaht et al., 2013; Chen et al., 2017; Tron et al., 2020; Franken et al., 2022). Trough concentration (C_0_) serves as a conventional surrogate index for assessing TAC exposure (Ghafari et al., 2019). It is valuable in the regulation of TAC dosing regimen. However, there are limitations of C_0_ in the estimation of TAC exposure, especially the influence of various factors on the TAC PK is not estimated. Through modeling and simulating based on results of TDM and patients’ characteristics, the individualized therapeutic regimen can be designed and adjusted. The *maximum a posteriori* (MAP) model, derived from the *a priori* population pharmacokinetic (PPK) model, proves valuable in formulating and regulating TAC dosing regimens. This approach offers the advantage of assessing and incorporating various factors into the PPK model to enhance predictive accuracy (Jing et al., 2021). While the MAP method excels in interpreting the mechanical characteristics of TAC PK data and accounting for inter- and intra-individual variations, it is noteworthy that individual trough concentration prediction errors may be relatively high due to inaccurate parameter assumptions or covariate effect modeling.

The use of machine learning (ML) in TDM and individualized therapy has rapidly developed in recent years. Its major advantage is the capability to process large amounts of data and explore the inherent characteristics of different data. ML can provide accurate predictions with a fast and efficient selection of covariates in large datasets (Fu et al., 2021; Sibieude et al., 2022). This method has been used to estimate the TAC concentration or dosage based on various factors, including body weight, age, pathophysiological status, concomitant drugs, and genetic polymorphisms of drug-metabolizing enzymes or transporters (Woillard et al., 2021). The ML method is suitable for predicting targets affected by many variables and sometimes shows stronger generalization and better accuracy (Huang et al., 2022; Song et al., 2023). Despite the higher accuracy of ML algorithms, there are some limitations to this strategy, such as inexplicable results (Destere et al., 2023). It can be assumed that a proper combination of two methods may provide more reliable predictions (Damnjanović et al., 2023).

This study aimed to establish a model of TAC ML combined with PPK in Chinese patients undergoing renal transplantation. The performances of different ML models and MAP in predicting the trough concentration of TAC were also compared.

## 2 Methods

### 2.1 Study design and population

A total of 127 adult Chinese renal transplant recipients who underwent their first renal transplantation were included in this study. The inclusion criteria for patients were as follows: i) primary renal transplant recipients meeting standard renal donor criteria and ii) administration of immunosuppressive drugs only after transplant surgery. The exclusion criteria included: i) combined organ transplantation, ii) panel reactive antibody positivity, iii) allergy or intolerance to TAC, and iv) pregnancy or lactation.

All patients received a postoperative triple immunosuppressive regimen consisting of TAC, mycophenolate mofetil (MMF), and steroids. TAC (Prograf, Astellas) was orally administered at 0.1 mg kg^-1^·day^-1^ twice daily, then adjusted to C_0_: 10–13 ng mL^-1^ in the first month and 5–9 ng mL^-1^ thereafter. A 1000 mg dose of MMF (Cellcept, Roche) was administered within 6 h before renal transplantation, and the same dose was given every 12 h after transplantation. Methylprednisolone (Pfizer, Puurs) was administered intravenously during surgery, progressively tapered, and then maintained at 5–10 mg oral prednisone daily after the first-month post-transplantation. Patients’ pathophysiological characteristics were collected on the day of TAC TDM. Demographic data, such as age, sex, and body weight (WT), and clinical data, including red blood cell count (RBC), hematocrit (HCT), platelet count, alkaline phosphatase (ALP), total bilirubin (TBIL), creatinine clearance (CLcr), and albumin (ALB), were recorded. Postoperative date (POD) was defined as the period between the day of the operation and the day of data collection. Blood samples were collected at 8:00 a.m., just before the morning dose. The collected data were randomly divided into training (80%) and testing (20%) datasets.

### 2.2 Determination of tacrolimus concentration and *CYP3A5* genotypes

Whole blood TAC levels were determined using the enzyme-multiplied immunoassay technique with the SYVA VivaEmit 2000 kit (Siemens Healthcare Diagnostics Inc., Erlangen, Germany). Whole blood (200 µL) was collected from patients for genotype analysis, and the detection range was 2–50 ng/mL.

Leukocyte DNA was extracted from peripheral blood samples using the TIANamp Blood DNA Kit (Tiangen Biotech Co., Beijing, China), following the manufacturer’s standard protocol. The CYP3A5*3 (rs776746) genotype was identified through PCR-sequencing. The primer sequences were as follows: CYP3A5*3: 3A5P1 (5′GCC CTT GCA GCA TTT AGT CCT T3′) and 3A5P2 (5′CCT GCC TTC AAT TTT TCA CTG 3′). The 50 μL reaction mixture contained 15–50 ng genomic DNA, 1 U of Taq DNA polymerase, 1× buffer, 0.2 mmol/L dNTP mixture, 1.5 mmol/L MgCl_2_, and 0.5 μmol/L of each primer. The reaction conditions were as follows: 7 min at 94°C, followed by 30 cycles at 94°C for 30 s, 60°C for 30 s, and 72°C for 30 s, with a final extension at 72°C for 7 min. The resulting product was purified and sequenced using an automated genetic analyzer (ABI 3730 Sequence Detection System; Applied Biosystems).

### 2.3 Population pharmacokinetic modeling

The PPK model was developed using the nonlinear mixed-effects modeling software NONMEM version 7.4.1 (Icon Development Solutions, Hanover, Maryland, USA), Pirana (version 23.1.1, Certara) and Notepad++ and PsN (Perl-speaks-NONMEM, version 5.3.1). R (version 4.3.0) was used in data processing and graphing. The first-order conditional estimation (FOCE) method was employed to estimate relevant parameters. Model selection relied on the objective function value (OFV), parameter estimates, and standard errors.

To ensure that random effects were distributed around zero, concentrations were log-transformed. Inter-individual variation IIV) of the parameters was modeled exponentially, while the residual error was analyzed additively to maintain variation within the same order of magnitude. The structural model is defined by the following equation:

$$P_{i}=TV\left(P_{i}\right)\times e^{\eta i\;}$$ (1)

$${lnC}_{obs}=\ln C_{pred}+\varepsilon$$ (2)

where P_i_ and TV (P_i_) are the individual and population values of the parameters described in the equation, respectively. η_i_ was the random error of P_i_. The values of η_i_ were assumed to be independently normally distributed with a mean of 0 and a variance of ω^2^. In the second equation, C_obs_ is the observed concentration, C_pred_ is the predicted concentration, and ε is the residual error with a mean of 0 and a variance of σ^2^.

Patient physiological and pathological characteristics, along with genetic polymorphisms, were assessed as potential covariates in the TAC PPK model. For categorical covariates including *CYP3A5* genotype, discrete numbers were given to each index: 0 and one for male and female patients. 0, 1, 2 for *CYP3A5* *1/*1, *1/*3 and *3/*3 patients, respectively. Both forward inclusion and backward elimination methods were employed in constructing the final regression model. Each candidate covariate was scrutinized by incorporating it into the baseline model, and weighted residuals, along with changes in the objective function value (OFV), were observed throughout the model-building process. Changes in the OFV approximate the χ^2^ distributions with degrees of freedom (df) equal to the number of covariates introduced. A covariate was deemed statistically significant if the OFV decreased by 6.63 or more (*p* < 0.01, df = 1) upon its addition to the base model during forward inclusion. The full model included all covariates that exhibited a significant decrease in OFV. Subsequently, each covariate retained in the model was eliminated by fixing its value to zero. This procedure was repeated until the value of the objective function failed to increase by 7.88 (*p* < 0.005, df = 1) (backward elimination). Individual PPK parameters, arithmetic means, and standard deviations were calculated using NONMEM Bayesian estimates from the POSTHOC output.

The test dataset was utilized to evaluate the accuracy and applicability of the final model and ensure its stability and predictive power. A visual predictive check (VPC) was conducted by simulating 1,000 datasets to assess the performance of the TAC PPK model. The distribution of concentration-time data for the simulated population (5%, 50%, and 95% quartiles) was compared with that of the original dataset to investigate the accuracy and predictive capability of the established model.

### 2.4 Machine learning models development and evaluation

A multilayer perceptron (MLP), support vector machine (SVM), and extreme gradient boosting (XGBoost) were utilized to develop the ML models. The indicators collected previously were included in the model: WT, AMT, POD, RBC, HCT, DBIL, BUN, CLcr, ALB, and the genotype of *CYP3A5*, while combinations of drugs were also integrated. The total unit dose per kilogram of body weight since the last blood concentration (UDOSE) was also calculated. Additionally, the individual concentration prediction (IPRE) of the TAC PPK basic model was tested.

To avoid biased performance estimates, nested cross-validation was performed using an inner 10-fold cross-validation for training and tuning, and an outer 10-fold cross-validation was used for validation after all training and tuning trials. The tuning process was performed using a Tree-structured Parzen Estimator (TPE), which employs the sum of the mean values of the mean absolute error (MAE) and mean absolute percentage error (MAPE). Each ML model pipeline was iterated 100 times within a predefined hyperparameter search space. The prediction performances in the training and tuning processes were evaluated using the R^2^ score, MAE, MAPE, and root mean squared error (RMSE). The equations are as follows:

$$R^{2}=1-\frac{{\sum_{i}\left({\hat{y}}_{i}-y_{i}\right)}^{2}}{{\sum_{i}\left(\bar{y}-y_{i}\right)}^{2}}$$

$$MAE=\frac{1}{n}\sum_{i=1}^{n}\left|y_{i}-{\hat{y}}_{i}\right|$$

$$MAPE=\frac{1}{n}\sum_{i=1}^{n}\left|\frac{{\hat{y}}_{i}-y_{i}}{y_{i}}\right|\times 100\%$$

$$RMSE=\sqrt{\frac{1}{n}\sum_{i=1}^{n}{\left(y_{i}-{\hat{y}}_{i}\right)}^{2}}$$

where $y_{i}$ is the actual value of TAC C_0_, ${\hat{y}}_{i}$ is the predicted value, $\bar{y}$ is the mean value. The highest R^2^ score and lowest MAE, MAPE, and RMSE indicated the highest fitting degree, and the best fitting result was used as the basis for the algorithm selection.

The performances of the MLP, SVM, and XGBoost models based on PPK were validated using the test dataset. The performance of the predictions based on the final PPK model was also validated. R^2^, MAE, MAPE, and RMSE were used as the performance indices. We also compared the prediction performance of various models among different TDM results.

## 3 Results

### 3.1 Patient characteristics

A total of 2041 concentrations from 127 renal transplant recipients were included in the model training (n = 103) and test (n = 24) sets. The patient demographics, laboratory data, concomitant medications, and genetic information of the training and testing datasets are shown in Table 1. This study included 81 male and 46 female patients, with an average age of 42.2 ± 11.0 years and weight of 62.6 ± 12.2 kg. TAC was administered between 3 and 1,622 days after transplantation. During therapy, 54.3% and 7.91% of patients received calcium antagonists and voriconazole, respectively. There were 9.45%, 33.9%, and 56.7% of patients with CYP3A5 *1/*1, *1/*3, and *3/*3 genotype, respectively.

**TABLE 1:** Patients’ demographic and clinical information and genotype of *CYP3A5*.

Characteristics	Training data set (80%)	Test data set (20%)
No. of patients	103	24
Age, years	41.2 ± 11.2	44.7 ± 8.91
Gender (Male/Female)	64/39	17/7
Hight, cm	168.1 ± 7.72	169.3 ± 7.12
Weight, kg	63.3 ± 12.9	62.6 ± 11.0
Post Operation Days	130 ± 231	175 ± 242
WBC, x 10^9^/L	9.15 ± 4.08	10.0 ± 4.51
RBC, x 10^12^/L	3.06 ± 0.61	2.98 ± 0.51
PLT, x 10^9^/L	150.6 ± 54.3	126.8 ± 53.5
ALP, mmol/L	58.2 ± 24.9	63.0 ± 44.6
TBIL, μmol/L	11.3 ± 4.49	11.8 ± 4.11
DBIL, μmol/L	2.58 ± 1.17	2.91 ± 1.45
BUN, mmol/L	18.8 ± 10.5	243.0 ± 14.4
CR, μmol/L	212.5 ± 208.5	305.5 ± 340.6
HCT	0.29 ± 0.056	0.27 ± 0.051
TP, g/L	55.6 ± 5.54	53.6 ± 5.06
ALB, g/L	34.0 ± 3.69	33.1 ± 4.04
Concomitant medication		
Calcium Antagonists	81 (78.6%)	21 (87.5%)
Proton Pump Inhibitors	102 (99%)	24 (100%)
Voriconazole	21 (20.4%)	8 (33.3%)
Genotype of *CYP3A5*		
*1/*1	10 (9.7%)	2 (8.3%)
*1/*3	35 (34%)	8 (33.3%)
*3/*3	58 (56.3%)	14 (58.3%)

### 3.2 Population PK modeling

As only C_0_ of TAC was used in establishing the TAC PPK model, a one-compartment model with first-order elimination was applied to the structural model. The value of k_a_ was fixed at 3.84 h^-1^. The clearance (CL/F) and volume of distribution (V/F) of the training data were 41.1 ± 13.8L/h and 2,620 ± 1,624 L, respectively. The PPK parameters of the structure and final model have been listed in Table 2.

**TABLE 2:** Population pharmacokinetic parameters of structue model and final model.

Parameters	Structure model			Final model		Test set final model	
	Estimates		RSE %	Estimates	RSE%	Estimates	RSE%
K_a_ (1/h)	θ1	3.86 (fixed)		3.86 (fixed)		3.86 (fixed)	
V/F L)	θ2	2,620	10.3	2,560	10.7	2,330	37.2
CL/F (L/h)	θ3	41.1	14.2	70.6	9.75	114	20.7
HCT on CL/F	θ4			−0.122	47.1	−0.161	31.2
CYP3A5 on CL/F	θ5			−0.348	12.2	−0.395	23.3
IIV							
ωV/F (%)	η_1_	73.6	21.5	65.0	22.5	69.6	33.1
ωCL/F (%)	η_2_	31.8	18.9	23.0	23.1	12.8	18.7
Residual error (%)	δ	37.7	12.4	35.6	12.0	33.3	21.6

The final model was formed after analyzing all covariates by forward inclusion and backward elimination. The HCT and *CYP3A5* genotypes (*1/*1, *1/*3, *3/*3 were set to 0, 1, and 2, respectively, and introduced in the model) showed significant changes in OFV when tested as covariates of CL/F. The final CL/F model is as follows:

$$CL/F=70.6\times e^{\left(CYP3A5\times -\;0.348\right)}\times e^{\left(HCT\times -\;0.122\right)}$$

**FIGURE 1:**
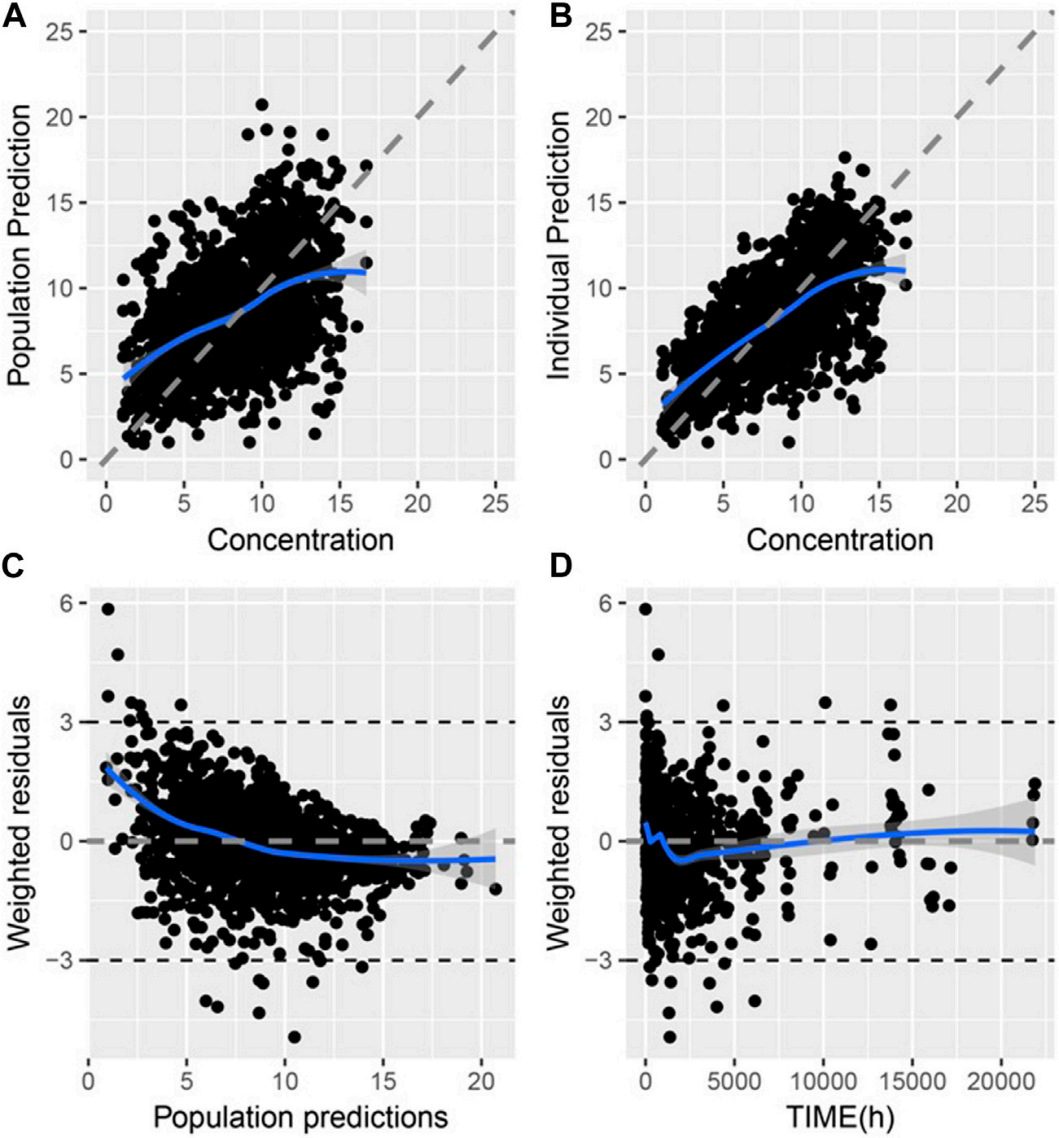
Goodness of fit of final PPK model of TAC in Chinese renal allograft recipients **(A)**. Population predicted concentration (PRED) vs. measured concentration (CONC); **(B)**. individual predicted concentration (IPRE) vs. CONC; **(C)**. Conditional weighted residual error (CWRES) vs. PRED; **(D)**. CWRES error vs. time.

The goodness-of-fit (GOF) of the final model is shown in Figure 1, where the population prediction (PRED) and IPRE correlated well with the measured concentrations.

**FIGURE 2:**
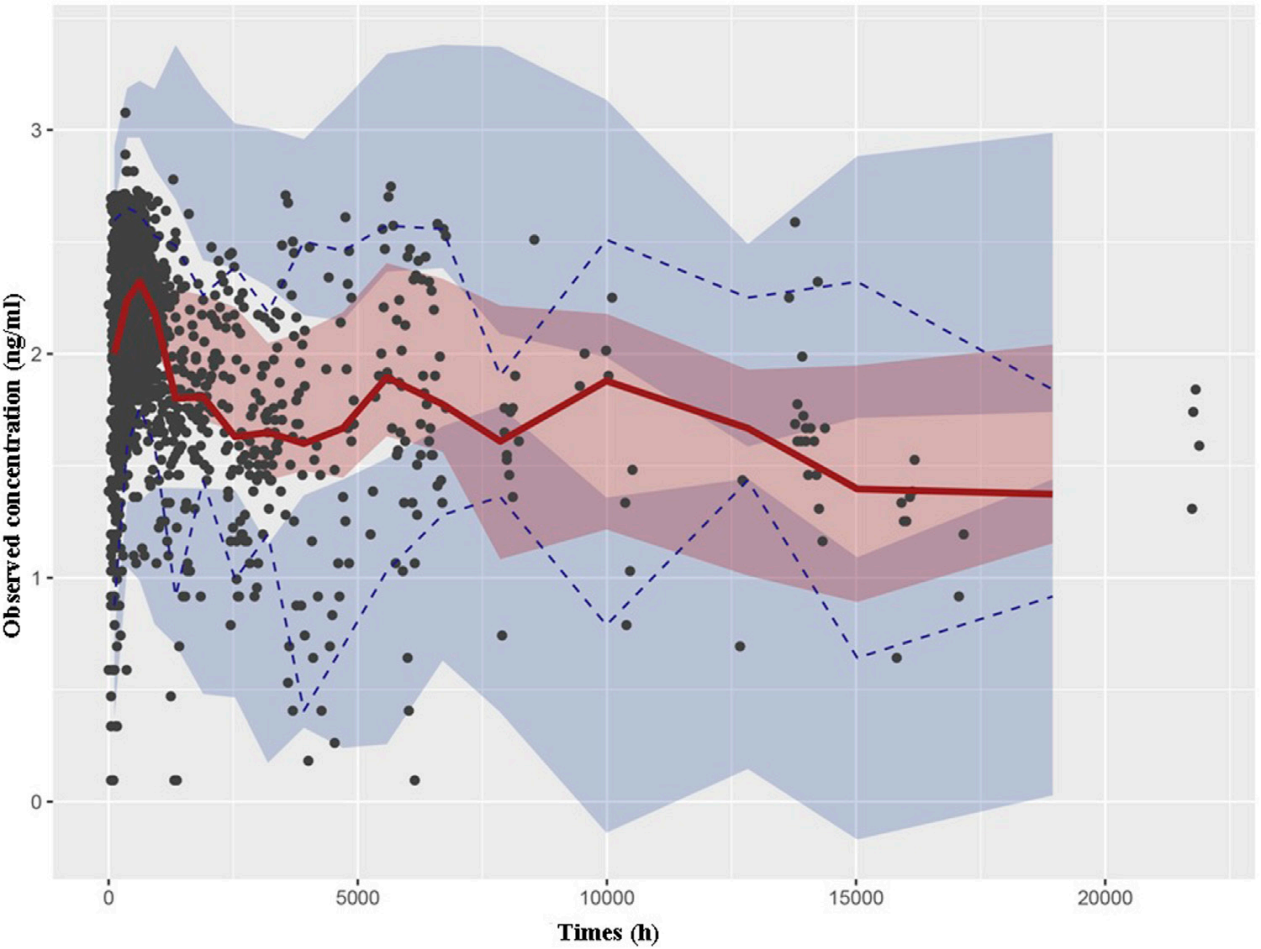
Visual predictive check based on the PPK model of TAC in Chinese renal allograft recipients. The solid lines represent the 50th observed data, the upper and lower dashed lines represent the 95th and fifth observed data, and the black solid cycles represent the observed data. Shaded areas correspond to simulated 95% confidence intervals.

Through a Bayesian estimation method, the individual predicted TAC concentrations of validation group (24 patients, 331 points) were compared with the observed data. The MPE (95% CI) was 1.23% (−1.67%, 4.12%), and the MRSE% was 23.7%. The bias was not significantly different from 0. The predictive performance of the final model was assessed through VPC in validation group (Figure 2). The predicted and actual values exhibited a significant correlation, with the majority of the measured TAC C_0_ values falling within the 95% CIs of the predicted concentrations.

### 3.3 Machine learning modeling

The MLP, SVM, and XGBoost algorithms of the ML model were developed based on various features, including *post hoc* prediction of the test dataset of PPK basic model parameters. The hyperparameters of the ML models were obtained from 100 TPE iterations using the training set. The optimal parameters of the ML model are listed in Table 3.

**TABLE 3:** Hyperparameters of machine learning model.

Model	Core hyperparameters
MLP	alpha = 0.007509; h_layer1 = 56; h_layer2 = 211; h_layer3 = 25; lr_init = 0.0003293
SVR	C = 9.244; degree = 41; gamma_svr = 0.014
Xgboost	colsample_bytree = 0.8357; esr = 36; eta = 0.061; gamma = 2.3214; max_depth = 7; min_child_weight = 0.72; n_estimators = 175; reg_lambda = 0.57835; subsample = 0.61

**FIGURE 3:**
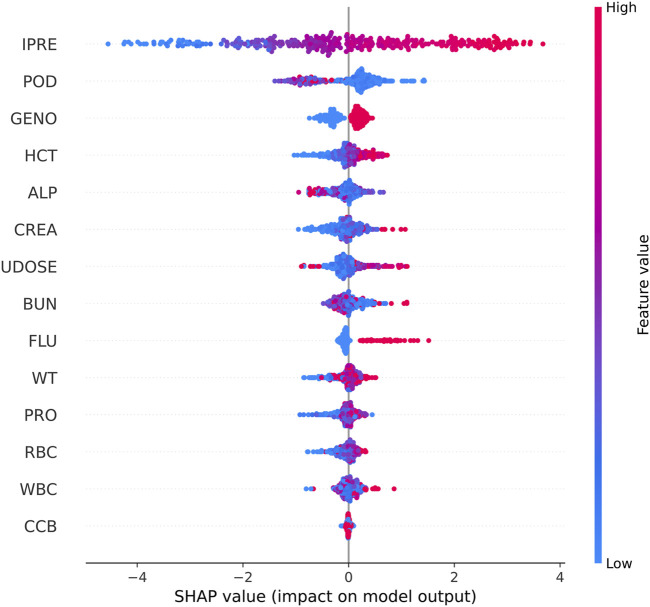
SHAP summary plot of features in the final model for the estimation of TAC in Chinese renal allograft recipients.

Shapley Additive Explanations (SHAP) was used to explain the model output (Figure 3). IPRE of the TAC PPK basic model, POD, *CYP3A5* genotype, and HCT were ranked higher than those of the other factors.

### 3.4 Comparison of predictive performances

**FIGURE 4:**
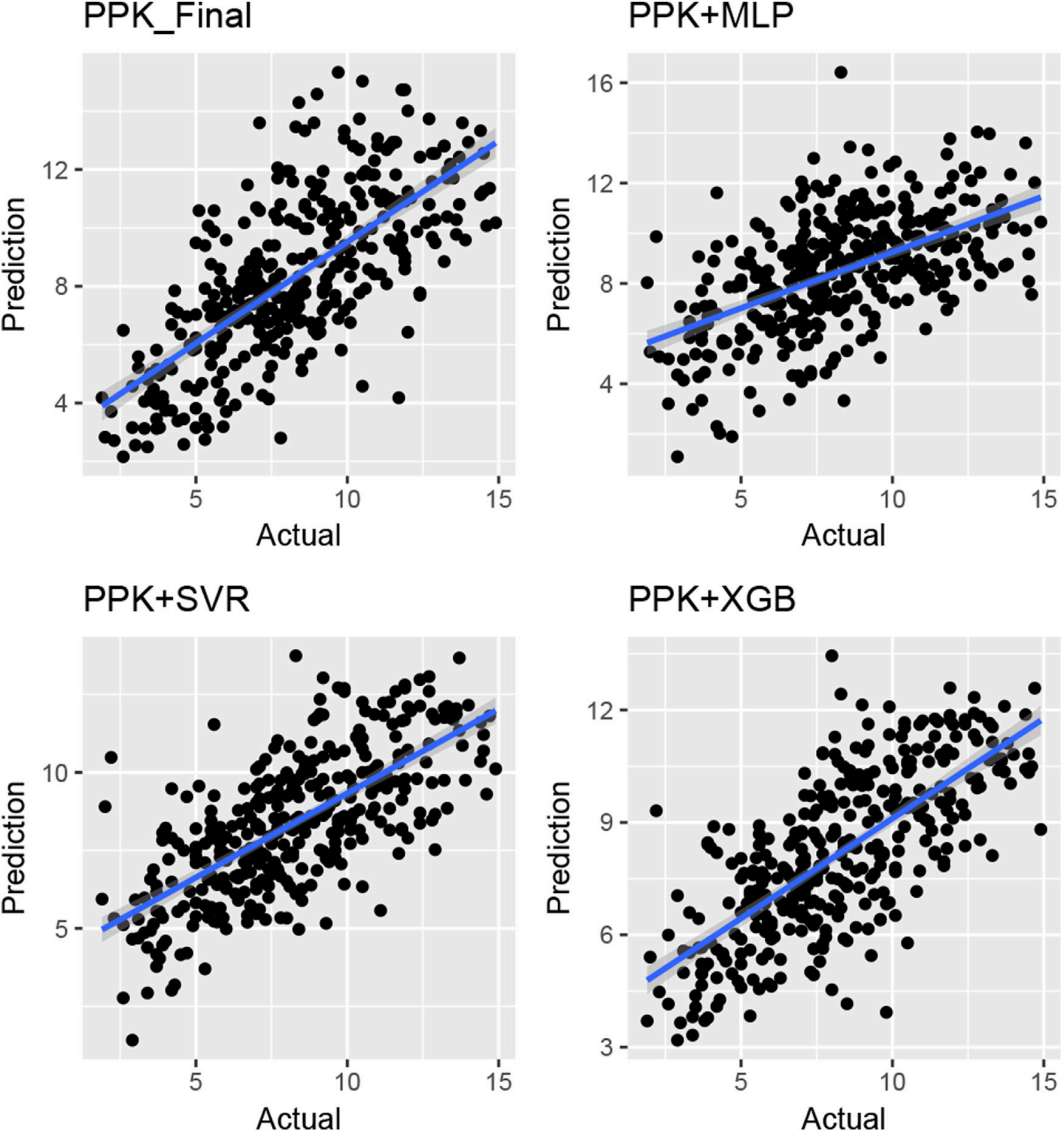
Plot of observed *versus* individual predicted TAC concentration by various machine learning models and Bayes estimation based on PPK model in the validation group of Chinese renal allograft recipients. (*n* = 328).

The TAC C_0_ prediction performed well in the test dataset using MAP based on the final PPK model, as well as with the MLP, SVR, and XGBoost models based on the basic PPK model (Table 4; Figure 4). Among these models, XGBoost, based on the PPK basic model, exhibited the highest performance (Table 4).

**TABLE 4:** Evaluation results of PPK model and machine learning models in test dataset.

Model	MAE	MAPE (%)	RMSE	R^2^ score
PPK Basic	1.85	26.9	2.44	0.46
PPK Final	1.77	25.3	2.35	0.48
MLP	2.04	30.5	2.54	0.25
SVR	1.67	25.8	2.12	0.48
XGboost	1.61	23.7	2.03	0.52

**FIGURE 5:**
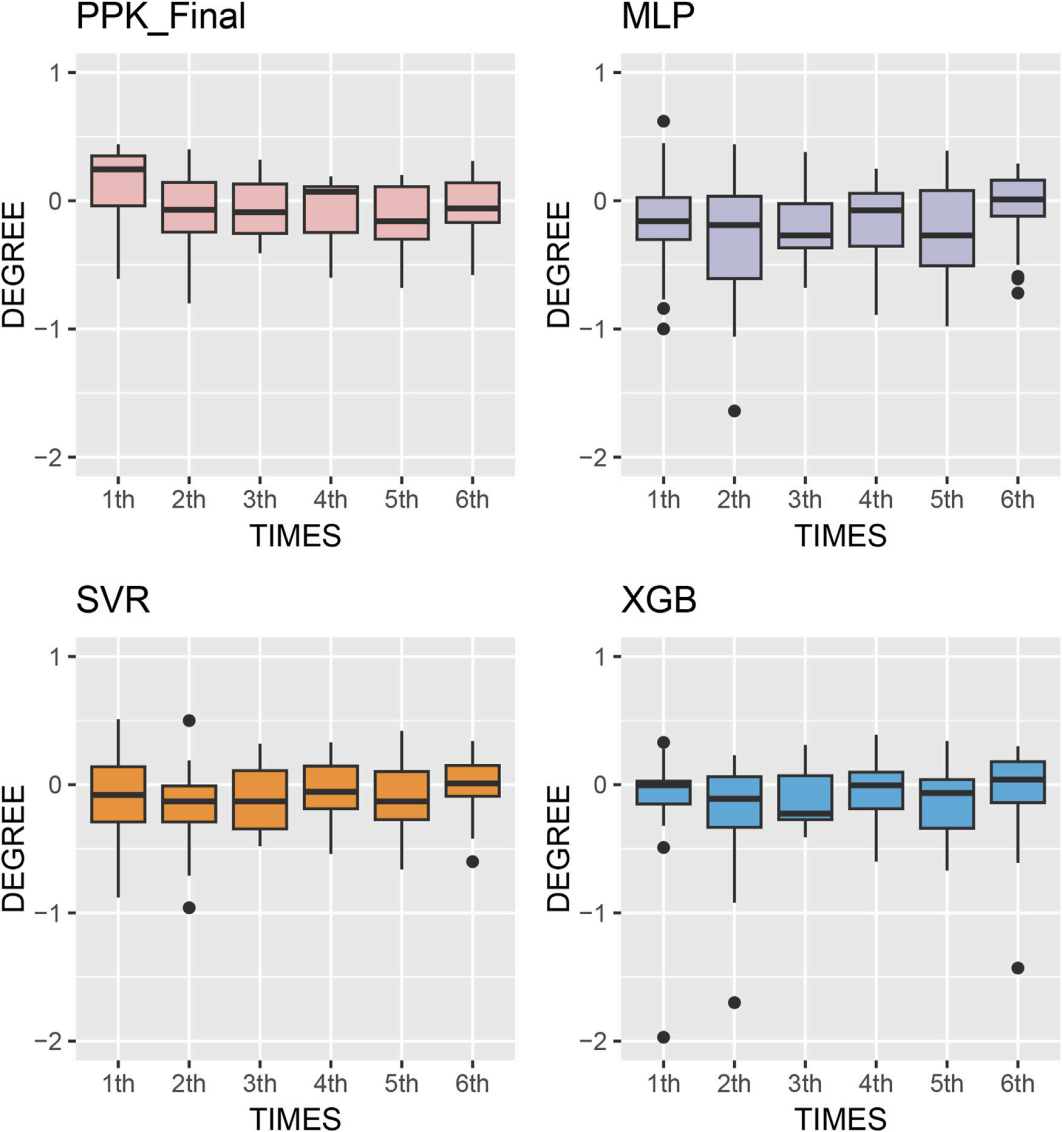
Box plots of prediction error with machine learning model and Bayesian forecasting in different scenarios (1st to sixth TDM results post transplantation).

The MPE of C_0_ at various time points after TAC administration was compared in test group. Starting from the second dose, the prediction errors from various methods tended to approach zero. Simultaneously, MAE and RMSE of the different methods showed significant improvement compared to the first administration, with XGBoost based on PPK demonstrating superior performance (Figure 5). This suggests that, by utilizing individual parameters and incorporating prior mechanisms such as inputting the IPRE, ML methods can effectively manage more covariates and complex effects, leading to reliable predictions through proper training and pipeline tuning.

## 4 Discussion

In this study, we developed an approach that combines PPK and ML models to enhance the prediction performance of C_0_ for TAC in Chinese renal transplant recipients. The PPK-based XGBoost model outperformed the PPK final, PPK-based SVR, and PPK-based MLP models in predicting C_0_.

Various PPK models have been established for different patient populations. The two-compartment model is commonly utilized as a structural model for PPK studies in patients following an intense sampling strategy (Zhu et al., 2014; Riva et al., 2023). Conversely, the single-compartment model is the most frequently employed PPK model based on conventional TDM data (Reséndiz-Galván et al., 2019; Teng et al., 2022). Approximately 60% and 40% of published models are one- and two-compartment models, respectively. In our previous study, we established a two-compartment TAC PPK model using rich-time PK data and conventionally monitored C_0_ data from Chinese patients. Based on this model, we estimated the Bayesian estimator of TAC AUC. We found that the two- and one-compartment models were suitable for intense PK and C_0_ data, respectively. The difference in CL/F obtained using the one- and two-compartment models was not statistically significant (Chen et al., 2017). The AUCs estimated using different models were comparable.

A number of factors have been reported to influence the PK of TAC. Campagne et al. found that among 63 published PPK models, *CYP3A5* genotype, HCT, POD, WT were the most commonly reported covariates. These covariates impacted TAC PK parameters and necessitated dosing adjustments to achieve similar drug exposure among patients (Campagne et al., 2019).

The CYP3A5*3 allele (resulting from the *6986A>G* mutation in *CYP3A5* intron 3) leads to a splice defect in the mRNA, resulting in the production of an unstable and nonfunctional CYP3A5 enzyme (Dai et al., 2001; Hsieh et al., 2001). The CYP3A5*3 genotype is widely accepted to significantly affect the CL/F (Zuo et al., 2013a; Bergmann et al., 2014; Han et al., 2014) of TAC. In the present study, we found that the CL/F of CYP3A5*1/*3 and *3/*3 patients were 70.6% and 49.9% of those with the *1/*1 genotype, respectively. Including the CYP3A5*3 genotype as a covariate of the CL/F of TAC resulted in an 8.85% decrease in interindividual variation in CL/F.

As TAC is highly bound to erythrocytes, HCT may reflect the level of unbound TAC and further affect CL/F. It has been reported that HCT is lower in the early postoperative period in renal transplant recipients and increases with the recovery of renal function (Han et al., 2013). In the present study, we found that HCT as a covariate decreased the inter-individual variation in CL/F by 6.21%.

The integration of AI technology into medicine has revolutionized the approach to medical data mining. Unlike traditional statistical methods, which often struggle to uncover the inherent characteristics of flat data, ML algorithms excel at processing vast and complex datasets without mechanistic assumptions. The accuracy and practicality of models can be continually optimized with increasing of the participant data. ML algorithms have found extensive application in clinical drug therapy, with numerous models developed to predict dosage or exposure (Fu et al., 2021; Bououda et al., 2022; Ponthier et al., 2022). Woillard et al. (Woillard et al., 2021) introduced the XGBoost ML model, leveraging two or three concentrations (pre-dose, and 1 and 3 h post-dosing), which underwent rigorous testing across six independent full-PK datasets from renal, liver, and heart transplant patients. Their ML models, integrating four covariates (dose, type of transplantation, age, and time between transplantation and sampling), demonstrated superior performance compared to the MAP model. In another investigation, Zhang et al. conducted a comparative analysis of various ML and deep learning algorithms for predicting TAC dosing regimens. They determined that the TabNet algorithm exhibited the highest performance. Noteworthy variables influencing the TAC daily dose in their final prediction model included the last TAC daily dose, last TAC therapeutic drug monitoring value, time post-transplantation, HCT, Scr, aspartate aminotransferase, weight, *CYP3A5* genotype, body mass index, and uric acid (Zhang et al., 2022).

In the present study, we established ML models based on the results of the PPK basic model and patient demographic and pathophysiological data. Utilizing SHAP analysis, we compared the impacts of various factors on the prediction performance of TAC C_0_ levels. The IPRE of the TAC PPK basic model, POD, *CYP3A5* genotype, and HCT ranked higher than those of other factors. Additional indicators such as ALP, CREA, previous TAC dosage, and BUN also influenced machine learning modeling algorithms to varying degrees. Although used in limited patients during therapy in certain stage of therapy, co-administered voriconazole was proved to be a factor influence the prediction of TAC concentration. Among these factors, only *CYP3A5* genotype and HCT were proved to be the covariates of TAC PPK model.

The model established in this study represents a pipeline that combines the advantages of PPK and ML. It serves as a complementary tool to address oversimplification and mis-specification of pharmacokinetic mechanisms due to mathematical modeling (Stankevičiūtė et al., 2023). Based on the IPRE from the basic PPK model, the XGBoost algorithm demonstrated better predictions compared to other algorithms, as well as the final PPK model, when applied to the data of the test group. This finding indicates a significant improvement in prediction performance. In the test group, the MPE of 2-6 TDM sessions were 13.1%, 11.6%, 11.2%, 11.5%, and 8.68%, respectively. Additionally, 68 samples (54.8%) exhibited a PE within the range of ±20% (Figure 5).

By leveraging the established model, previous dosing regimens, and TDM results, regulated dosages can be estimated. Each dose can be paired with another index to simulate C_0_, allowing for the selection of the optimal dose regimen.

By predicting the concentration after the next dose, clinicians can gain a clear understanding of dose exposure. They can then judge whether the concentration falls outside the therapeutic range to adjust the dose accordingly, thereby enhancing control over the patient’s drug exposure. This adjustment can lead to a reduction in adverse effects and minimize the risk of graft loss. We also explored labeling dose-exposure data to predict the direct dose amount and provide dosage recommendations, although further research is necessary in this regard.

This study has certain limitations. Firstly, only the C_0_ of TAC was considered, and TAC concentrations at other time points may offer additional insights into pharmacokinetics. Secondly, other factors such as genetic polymorphisms of transporters and other co-administered drugs (i.e., Wuzhi capsules) could potentially influence prediction accuracy. Thirdly, this study is a single-center study, while the data were split into training and test groups, validation with data from other centers could provide more robust validation for the established model.

## 5 Conclusion

In summary, we utilized PPK model-based machine learning algorithms to develop a TAC concentration prediction model tailored for Chinese renal transplant recipients. Through comparison with various algorithms and the MAP method, we found that the PPK model combined with XGBoost yielded superior prediction performance. The model developed in this study offers a promising avenue for designing personalized TAC dosing regimens for Chinese patients undergoing renal transplantation.

## Data Availability

The original contributions presented in the study are included in the article/Supplementary Materials, further inquiries can be directed to the corresponding author.
