# Supplementary material for: Improving prediction of tacrolimus concentration using a combination of population pharmacokinetic modeling and machine learning in chinese renal transplant recipients
Source: Front Pharmacol. 2024 May 9;15:1389271. doi: 10.3389/fphar.2024.1389271 (PMC11111944; doi:10.3389/fphar.2024.1389271)
Supplement: Supplementary file 2 [file DataSheet1.docx]

Supplementary Material

# Supplementary Figures


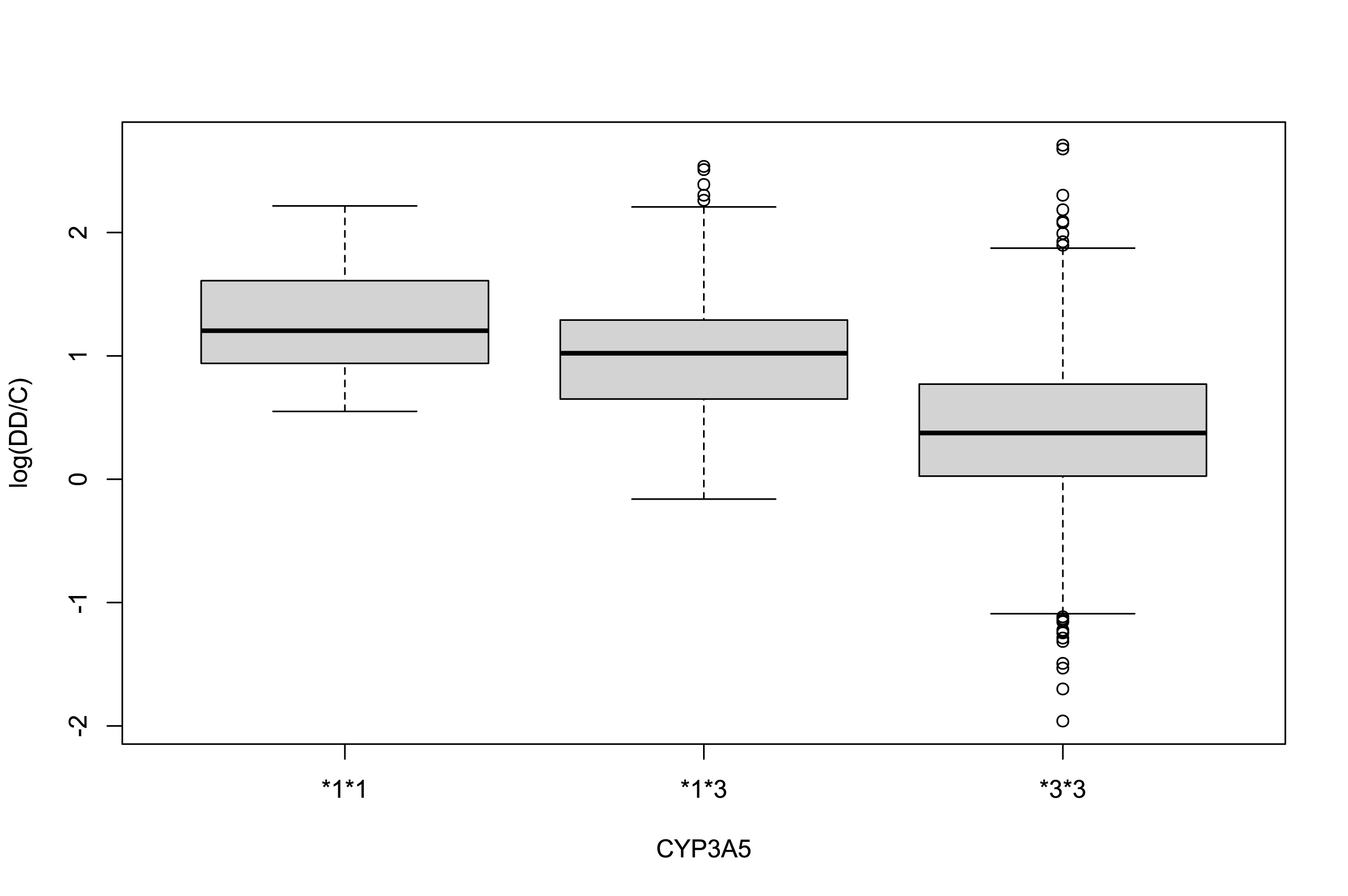


**Supplementary Figure1**. Box plots of log-transformed daily dose-blood concentration ratios (DD/C) between different CYP3A5 genotypes of all patients.


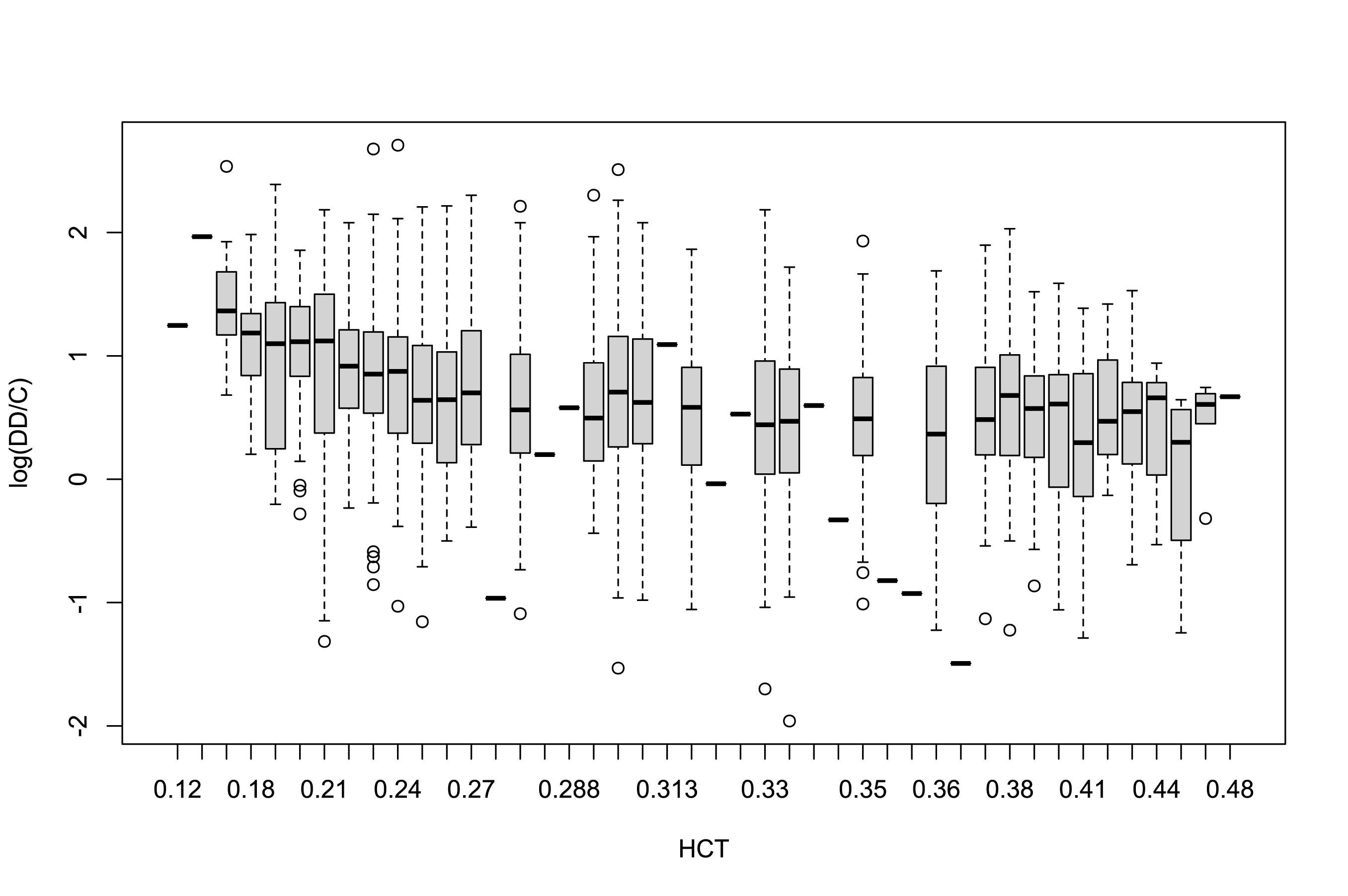


**Supplementary Figure 2**. Box plots of log-transformed daily dose-blood concentration ratios (DD/C) in HCT of all patients.


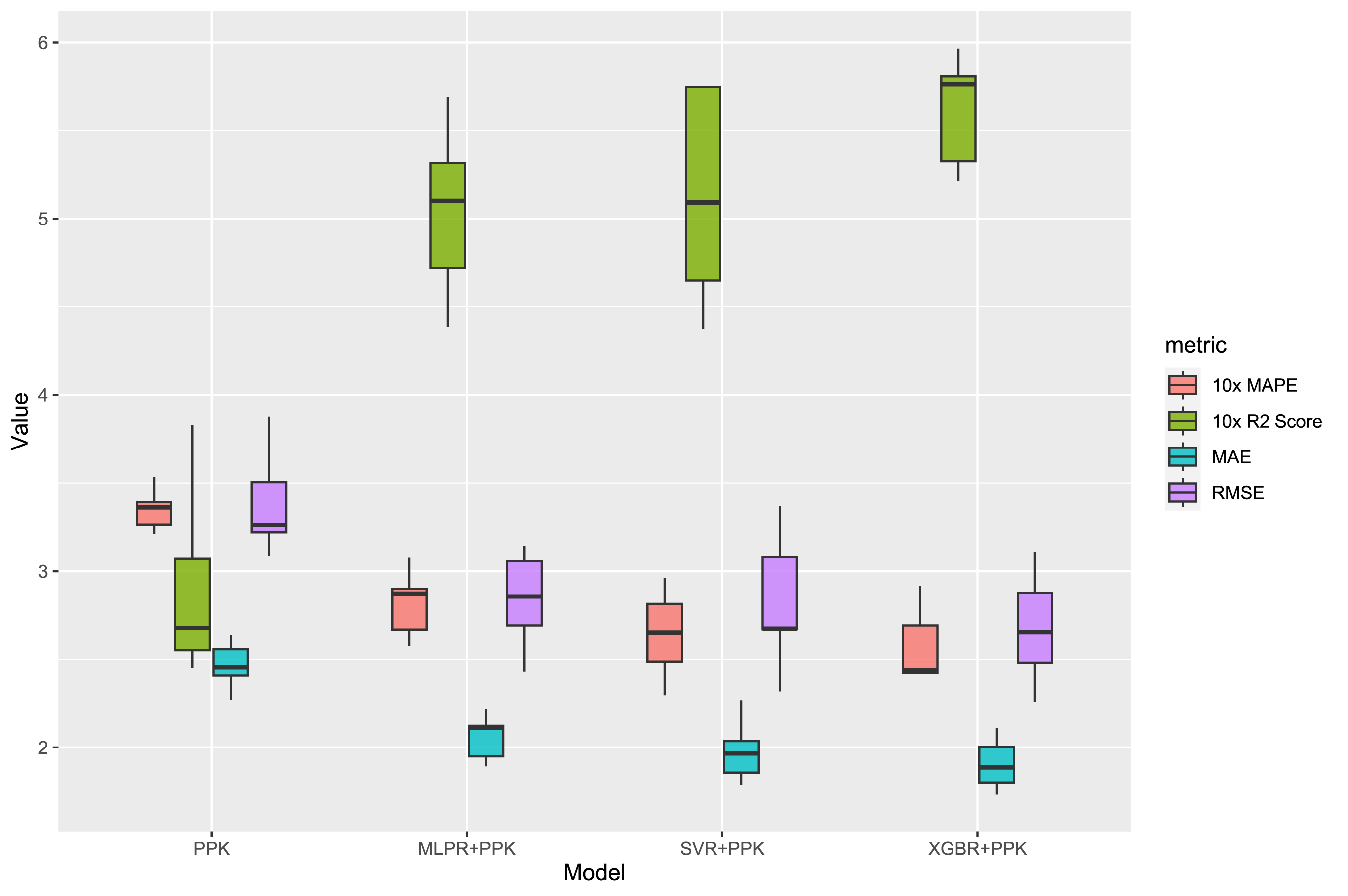


**Supplementary Figure 3**. Comparison box plots of predictive performance of five prediction models
